# Success Factors for Scaling Up the Adoption of Digital Therapeutics Towards the Realization of P5 Medicine

**DOI:** 10.3389/fmed.2022.854665

**Published:** 2022-04-12

**Authors:** Alexandra Prodan, Lucas Deimel, Johannes Ahlqvist, Strahil Birov, Rainer Thiel, Meeri Toivanen, Zoi Kolitsi, Dipak Kalra

**Affiliations:** ^1^empirica Gesellschaft für Kommunikations- und Technologieforschung mbH, Bonn, Germany; ^2^Finnish Innovation Fund SITRA, Helsinki, Finland; ^3^The European Institute for Innovation Through Health Data, Ghent, Belgium

**Keywords:** digital therapeutics, P5 Medicine, scaling up, adoption, success factors, assessment, certification, regulation

## Abstract

**Introduction:**

Digital therapeutics (DTx) can be a valuable contribution to the successful scale up of P5 Medicine (personalized, participatory, predictive, preventive, precision medicine) as they offer powerful means of delivering personalization and active patient participation in disease self-management. We investigated how the approval and adoption of DTx within health systems have been approached in five selected European countries and regions, with a view to proposing success factors scaling up their adoption.

**Methodology:**

Preliminary research established best countries or region candidates as being Germany, UK, France, Belgium, and the Spanish Region of Catalonia. The research was informed by a literature review, interviews with public bodies and industry, and a multi-stakeholder workshop to validate the findings and fill in existing gaps.

**Results:**

To authorize the use of digital technologies, the countries and regions passed legislation and developed policy instruments, appointed bodies to assess and certify the products and formalized mechanisms for permitting reimbursement. While DTx is not a commonly used nomenclature, there are digital health technology types defined that have similar requirements as DTx. Assessment and certification frameworks are usually built around the Medical Device Regulation with additional criteria. Reimbursement considerations often observe reimbursement of therapeutic devices and/or medicines. To be integrated into reimbursement systems, countries require manufacturers to demonstrate clinical value and cost-effectiveness. As there are currently very few DTx approved in practice, there is resistance toward clinical acceptance and organizational change, and change management is highly needed to integrate DTx into healthcare systems. The integration and secondary use of DTx data is not encountered in daily practice. Although some enablers exist, there remain technical and legal barriers.

**Discussion:**

DTx strategies should be considered as an integral part of digital health strategies and legislation, and specific DTx pathways with clear and transparent assessment and guidelines that balance regulation and innovation should be defined. To help manufacturers, countries should recommend and list methods that are widely accepted and ensure scientific robustness, aligned to the MDR requirements to support transfer of relevant and comparable data across countries. To facilitate rapid uptake of innovation, countries should add flexibility to the framework by allowing temporary market authorization to enable data collection that can support the clinical and socio-economic evaluation and data gathering phase. Certification should trigger rapid price setting and reimbursement mechanisms, and dynamic ways to adjust price and reimbursement levels in time should be established. Relevant stakeholders should be approached on the potential impacts of DTx through transparent communication and change management strategies should be considered. These findings should be validated with a wider range of stakeholders.

## Introduction

The digital transformation of society comes about at different speeds, depending on the observed sector. Healthcare is traditionally delivered in-person; however, digital support tools are increasingly relied upon during different phases of care (e.g., diagnosis, communication, treatment) (1, 2). Electronic Health Records (EHRs), e-prescriptions and e-referrals are only a few examples of services and tools aimed to digitize healthcare. This development is further strengthened by the recent emergence of digital therapeutics (DTx). Sometimes referred to as “apps on prescription,” DTx are regulated digital, and often, mobile applications that deliver evidence-based therapeutic interventions to either prevent, manage, or treat a disease (3–6).

Digital therapeutics can be a valuable contribution to the successful scale up of P5 Medicine (medicine that is personalized, participatory, predictive, preventive and palliative). As argued by Blobel et al. (see the first paper in this volume), digital transformation (in partnership with organizational and workflow transformation) is essential to realizing this vision (7). Digital tools such as apps, wearables, and sensors, especially those that offer active guidance to patients on personal actions, escalation actions and treatment dosing, offer powerful means of delivering personalization and active patient participation in illness self-management. As they are not intended to replace existing therapies, digital therapeutic solutions are often used in combination with medications, other devices or therapies and are mainly targeted at patients as the users (3). They are therefore adopted as part of a care plan, through a joint decision by clinicians and patients and ideally as a fully integrated component of the plan, embedded within the health and care system.

As DTx do not fall within the scope of wellness and lifestyle apps, manufacturers undergo regulatory approval processes in order to receive marketing authorization that enables their adoption by health systems (4). Approved DTx may be prescribed by healthcare providers or procured on a larger scale (8, 9). However, two aspects are key to support a wide-spread and swift adoption of DTx into routine care: firstly, care providers need to be aware of the therapy and its ambition and, secondly, regulators need to implement reimbursement support for patients and healthcare providers in case interactions are part of the therapy (6). Potentially, DTx will introduce major changes to the accessibility of care for patients and their health outcomes (10, 11). This will, however, depend on the successful demonstration of their clinical and economic value proposition compared to existing interventions. Experts point out that low-price technology interventions do not necessarily trigger cost-savings, but in fact increase their demand and thus overall healthcare spending (12, 13).

Despite positive evidence, successful implementation and scaling-up of digital health solutions still seems to be a sluggish process and remains a much debated topic, with a very fragmented landscape (e.g., the fragmentation of national EHRs and ePrescription services, health data silos). Adoption of digital health technologies takes place across many dimensions of the health and care system and within diverse organizational processes. The enablers and success factors for adoption therefore need to be studied from a plethora of stakeholder and dimension perspectives.

Frameworks for scaling up digital health interventions have been proposed. For example, Yamey (14) analyses success factors for scaling up global health interventions. These include “choosing a simple intervention widely agreed to be valuable, strong leadership and governance, active engagement of a range of implementers and of the target community, tailoring the scale-up approach to the local situation, and incorporating research into implementation” (15). Labrique et al. (16) identified five key areas critical for the success of scaling digital health in low and middle-income countries. These comprise the initiative addressing unmet needs and offering tangible benefits, stakeholder engagement to implement new initiatives, a technical profile driven by simplicity, interoperability and adaptability, alignment with broader health care policy, and sustainable funding to support long-term growth (16). Desveaux et al. (17), approached the issue of digital health implementation from a policy perspective. To overcome policy-level barriers, they identified several key areas, that include the need for a system-level definition of innovation, a clear overarching mission, and clearly defined organizational roles. Operationally, the authors identified a need for standardization of processes, a shift in emphasis of change management, and alignment of funding structures (17).

A study that examined barriers and facilitators to the implementation of digital health at scale through the evaluation of a national digital health programme in the UK identified three levels of issues influencing the readiness for digital health: the macro-level (market, infrastructure and policy), meso-level (organizational), and micro-level (professional or public). Clinical endorsement, champions who promoted digital health and public and professional willingness were identified as factors that support implementation of digital health (18). Another recent study from 2022 examined key considerations for adoption and implementation of digital health tools within large, complex health systems (19). These were aimed to support health systems' decision-making on how to best approach the selection and evaluation of digital health tools, how to ensure the availability of sufficient resources for deployment and long-term use and the creation of implementation strategies. The dimensions described include optimal product selection, how clinical value and return on investment are demonstrated, internal champions, tool alignment with institutional priorities, executive sponsors, data assets, long-term operational anchoring and implementation-required resources.

Perspectives from a stakeholder workshop in Switzerland identified a culture of innovation and patient-centric approaches as a push factor, but that adoption was hindered by fear of change and unwillingness to share data (20).

Key success factors for policy-makers to consider when using demand-driven open innovation as a policy instrument involve improved citizen centricity through clinical staff engagement, promoting knowledge transfer through better and more communication between health system actors, time to market entry, customer relevance and making explicit to stakeholders process roles, responsibilities and funding structures (21). Another paper that explored success factors scaling-up digital innovations in healthcare pointed out that actors and factors on different levels influence success factors (micro, meso, macro and technology/innovation level) (22). The authors highlighted the importance of leadership as a trigger for innovation, a culture for change, common goals for change, interdisciplinary co-creation of solutions that address the needs for change through innovation from multiple perspectives, and the need for sound regulation and actions to maintain or increase trust in scaled-up solutions (ibid.).

However, none of these papers consider DTx specific-success factors and remain in the general digital health domain. As DTx are a rather novel form of therapy, a few countries have implemented DTx-specific assessment frameworks in addition to regulatory compliance with the Medical Device Regulation (MDR). The success factors for DTx adoption are therefore likely to be a combination of the success factors for obtaining approval and some that are the same as for any other digital health intervention. This topic has not been investigated to date. This paper explores how the approval and adoption of DTx within health systems have been approached in five selected European countries and regions, with a view to proposing success factors for scaling up their adoption.

## Methodology

The analytical framework was established in the beginning of the investigation and covered eight dimensions: system-wide strategic policies, the legal scope and nomenclature of DTx, assessment and certification schemes, clinical and socio-economic evaluation, integration into reimbursement systems, integration into healthcare systems, data integration and use of DTx and secondary use of data and data reusability. These eight dimensions were derived by examining the main categories of criteria within multiple European assessment frameworks (15), in the context of the authors' background knowledge of the general digital health success factors summarized in the previous section.

To ensure that all dimensions were covered, prior research was conducted to assess European countries in terms of availability of DTx assessment frameworks, certification and reimbursement approaches and number of approved DTx. Five countries or regions were identified to be most advanced in the field of DTx: Germany (specific DTx legislation, a clear “fast-track” certification and reimbursement scheme in place, several DTx solutions with both permanent and preliminary market authorization), Belgium (mHealth Belgium initiative and strategic focus on mHealth, pilot projects on DTx to determine appropriate framework for DTx integration, three-tiered validation approach), France (certification and reimbursement process for connected medical devices (CMD) based on a registry of procedures and services, with a guide for, or specific features of, clinical evaluation of a CMD in view of its application for reimbursement), National Health Service (NHS) England (several innovation and digital health technology frameworks on required evidence for reimbursement negotiations for health apps with Clinical Commissioning Groups and NHS Trusts) (23–29), and the region of Catalonia (existing certification framework for health and wellbeing apps, mConnecta platform is an interoperable infrastructure that integrates mobile data from mobile apps, wearables and medical devices with the EHR data) (30, 31). Data from this prior review was fed back to the finalization of the analytical framework. A mixed-methods approach was employed to facilitate the analysis. Firstly, the collection of information involved a systematically approached desk research of policy instruments, websites, templates, and guidelines. Secondly, 15 semi-structured interviews were conducted with at least two interviews from each of the five countries or regions, involving at least one expert from public authorities and one from industry. Experts were identified through internet search: public officials were contacted through the national bodies responsible for DTx assessment and certification, and industry experts were contacted through contact forms on companies' websites which produce and market DTx in that country or through public workshop documents (list of speakers or attendees). Thirdly, findings from the literature and the interviews were validated during a dedicated multi-stakeholder expert workshop, whose attendees received a summary of all collected information. At the workshop, the interim findings were presented, discussed, and remaining gaps were filled to the extent possible. The discussion was organized around four main areas: (1) evaluation and assessment, (2) reimbursement and procurement, (3) European alignment, and (4) secondary use of DTx data. The workshop hosted 25 experts from industry, public authorities, EU-initiatives, and networks and represented experts from all studied countries. The workshop input validated and consolidated the results across all methods and countries under the analytical framework. Furthermore, key discussion points facilitated the identification of success factors for enabling better integration of DTx into healthcare systems.

## Results

### System-Wide Strategic Policies

To authorize the use of digital technologies within the health system, the five countries and regions have passed legislation and developed policy instruments, appointed bodies with authority to assess and certify the products, and formalized mechanisms for permitting reimbursement. Countries such as Belgium and France included policy on DTx (referred to as connected medical devices or CE-certified mHealth apps) as part of a broader eHealth or digital health strategy. The Belgian national e-Health Action Plan 2013–2018 contains the general strategy of the architecture of the national health data platform (24). In the context of the plan, the Belgian authorities developed a dedicated mHealth assessment process following an assessment pyramid model (26). In France, DTx are an integral part of the “National Health Strategy 2022” and associated digital transformation, which promotes health reform measures, the reinforcement of governance, security, and interoperability, and the stimulation of innovation in digital care provision (32). NHS England created specific programs to enable rapid uptake of digital innovations (e.g., Accelerated Access Collaborative Programme, Medical Technologies Evaluation Programme, NHS Innovation Accelerator and Digital Health London), through which DTx adoption is supported in order to achieve common health policy goals such as cost reductions and improved quality of care (33–36). Germany does not have an overarching strategy to digitize the health and care sector, but rather particular laws to create the legal basis for digital innovation. The legal basis for reimbursement of digital health applications was established through the 2019 Digital Health Care Act (Digitale-Versorgung-Gesetz), which states that insured persons in the statutory healthcare insurance system are entitled to healthcare through digital health applications (33). The autonomous region of Spain, Catalonia, has defined via the 2015 Catalan Master Plan a Strategic Plan and an Action Plan to support the development of mHealth in Catalonia, through which it addresses certification and integration of mHealth apps, yet currently does not have policies to enable reimbursement of digital health applications (31).

### The Legal Scope and Nomenclature of DTx

“Digital therapeutics” is not a commonly used nomenclature in European legislation and policy. Different terms are used to refer to DTx in the five explored countries and regions: connected medical devices, digital health applications, digital health technologies, or mHealth apps. Variations can also be observed in the exact scope of what types of DTx are covered by legislation. Commonly found scoping criteria for inclusion of DTx into the relevant legislation were that they should be digital, have a patient-facing interface, address prevention, management or treatment of a medical disorder or disease, and possibly undertake analytic processing besides simply data collection and display. General health and wellbeing apps were not addressed by the studied countries, except Catalonia. In France, DTx fall under the category of medical devices and apps are classified to assist with clarifying which level is in scope of the legislation and approval process (29). The Belgium framework considers mHealth applications that are CE-marked as medical devices of all classes (37). According to NICE (the Evidence standards Framework for digital technologies—ESH) (28), in the UK DTx fall under the category of digital health technologies (38). Germany has defined DiGA (Digitale Gesundheitsanwendungen—Digital health apps)—a medical device of the MDR risk class I or IIa, whose main function is based on digital technologies achieving the medical purpose, where “DiGA supports the recognition, monitoring, treatment or alleviation of diseases or injuries and represents a “digital assistant” in the hands of patients” (23). Generally, inclusion of prevention was not explicitly stated but secondary and tertiary prevention for a particular disease was widely included. Primary prevention might be partly included in specific cases but mostly it is not considered to fall within this context.

### Assessment and Certification Schemes

All five explored countries and regions apply Health Technology Assessment (HTA) processes for digital health solutions that fall under the MDR. There are several types of assessment frameworks that a DTx solution can undergo: DiGA frameworks, frameworks for CE-marked health apps, classical HTA evaluation approaches for medical devices, frameworks for digital technologies, and frameworks for general health and wellbeing apps. While safety aspects and clinical effectiveness are partly ensured by certification as CE-medical devices under the new MDR, all five explored countries and regions have additional requirements related to risk assessment, safety evidence, data protection, health outcomes impact, or health economic implications (39). Germany is at the forefront of DTx assessment with its DiGA assessment process (23). The Digital Health Applications Regulation (DiGAV) describes the regulations and requirements for testing the eligibility of DiGAs for reimbursement by the statutory health insurance system (40). The Federal institute for Drugs and Medical Devices BfArM (Bundesinstitut für Arzneimittel und Medizinprodukte) is the responsible body for the evaluation and certification of DiGAs. From the moment of application, BfArM is obliged to perform the assessment within 3 months. In case of acceptance, the application is published in a specific DiGA directory (41). The procedure is designed as a fast track: if positive effects on care evidence are not available, then the DTx is preliminary listed and the evidence can be submitted within the next 12 months, with a further extension of maximum 12 months if justified. NHS England has a series of indicator frameworks for digital technologies (soft regulations) designed by NICE and NHSx—the Digital Technology Assessment Criteria (DTAC) (42). In the pyramid-based Belgian framework and certification process, at each of the three pyramid levels the DTx is evaluated for certain criteria by different institutions (26). In France, health technologies and medical devices are evaluated by the “Medical Device and Health Technology Evaluation Committee” (CNEDiMTS, part of Haute Authorité de Sante, HAS) according to internal assessment guidelines for medical device (29). The TICSalutSocial foundation, part of the Catalan Ministry of Health, created the “Accreditation Service and TICSS guarantee certification” framework through which a set of criteria was established for general health and wellbeing apps, but where the CE-mark is currently considered optional (43).

### Clinical and Socio-economic Evaluation

The evaluation of socio-economic and clinical evidence in Germany, France, Belgium, and the NHS England feature typical Health Technology Assessment (HTA) elements, including security, safety, and effectiveness.

The German DiGA assessment introduces the concept of positive care effect, which is split into two categories: medical benefits and patient-relevant improvements (5). Both categories refer directly to the patient and need to be demonstrated by appropriate endpoints (e.g., morbidity, mortality, or QoL). The positive care effects need to be demonstrated through clinical studies that show positive effect with a comparison group through controlled trials or randomized controlled trials. If sufficient evidence for a positive healthcare effect does not yet exist but all other requirements are fulfilled, the DTx company can apply for a provisional listing in the directory, as described earlier. The French CNEDiMTS published a “Guide to the specific features of clinical evaluation of a connected medical device (CMD) in view of its application for reimbursement” in January 2019 (44). The evaluation is currently built around Medical Device assessment and split into two stages: clinical value and real-world results. In the French evaluation procedure, a randomized clinical trial (RCT) is the preferred form of clinical evidence although lower level of clinical evidence can be submitted. The current model is based on a committee-based approach which makes case-specific decisions possible (45). The UK frameworks have different focus areas: the DTAC by NHSx focuses on technical questions while ESF by NICE describes clinical and socio-economic efficacy requirements, where clinical data needs to be acquired through experimental and comparative studies (28–42). The Belgian model is built around the MDR which requires clinical effectiveness evidence (46). The framework also considers changes to current care processes, costs, and clinical evidence. However, the specifics of the evidence is left open and many kinds of methods (e.g., RTCs, studies based on real-world use, or expert opinion) can be applicable, and the model leaves most room to maneuver for the applicant compared to other countries. The Catalan TICSS framework does not capture clinical or socio-economic evaluation of health apps (47).

Manufacturers in all countries are expected to support the costs of clinical studies. Randomized Clinical Trials are an expensive and time-consuming undertaking and smaller companies may not always be able to provide this gold-standard of clinical evidence. The investigation revealed that DTx companies' developers suffer from the lack of recognition of excellence, and they welcome assessment and evaluation, but processes must be efficient, realistic, and transparent. Much of what needs to be assessed is performed under regulatory MDR compliance. The main challenge from the manufacturers' point of view regarding clinical and socio-economic evidence is to have realistic requirements in terms of the evidence they must present. One challenge relates to the definition of health benefits and corresponding evidence: should it be clinical outcomes, improvements in the process of care or both? The latter can be challenging because it involves organizational change as well as an organizational adoption of the DTx. While pilots of DTx are feasible, it is harder and expensive to provide large-scale evidence. Therefore, there is need of a reasonable and sufficient level of population evidence that can demonstrate a realistic level of outcome-change based on a probably cautious organizational commitment to an unapproved DTx. Since healthcare systems are under increasing pressure for cost savings, many countries emphasize cost savings instead of added value.

The investigated countries and regions varied in the extent to which the expected standards for approval were openly available with the assessment criteria that a DTx developer needs to meet, but companies we interviewed valued having access to the most complete and precise guidance they could obtain to help them to submit the evidence that would be required. The evidence generated within the country in question is considered the gold standard and using data from another country requires clarification and reasoning from the developer.

### Integration Into Reimbursement Systems and Market Stimulation

DTx solutions can be commercialized through licensing agreements with hospitals, companies, or individuals, after being certified as CE-devices and proving clinical effectiveness through RCT studies. Reimbursement is a strong incentive, and it always requires that the DTx is prescribed by a health professional and being selected among solutions that have had a successful prior positive HTA-type assessment. However, approval and reimbursement are usually separate decisions, sometimes made by separate bodies based on separate applications, and an approved DTx could be used by a healthcare provider if it perceives a clinical and business case (without reimbursement). A clear link between certification and reimbursement has been defined for the German DiGA and mHealthBelgium frameworks. The two frameworks employ a bottom-up approach, where application is open for all DTx solutions. The ones that are certified under the specific assessment process are listed in a directory and reimbursed. In France, NHS England and Catalonia, there is currently no direct link between certification and reimbursement.

The German model of reimbursement for DTx is registry-based. After the solution is certified and approved by BfArM, price negotiations are conducted and established between manufacturers and the National Association of Statutory Health Insurance Funds (GKV-SV) (48). BfArM plays a consultancy role and informs the GKV-SV of the need for corresponding remuneration amount. In the first year, the manufacturer is free to set their price for that year according to value-based pricing principles and market competition (i.e., intensity of positive healthcare effects, preliminary manufacturer price, solution pricing in other countries). After the first year, the price setting is determined with a framework agreement designed by the GKV-SV. Reimbursement for DiGAs is currently part of a special budget but will become part of a budget that is allocated to primary care at regional levels. Besides the DiGA prescription, General Practitioners (GPs) can be reimbursed for additional services related to DiGA. The price negotiations in the future will show how attractive the market will be, and eventually determine the long-term success of the DiGA-framework.

In Belgium, solutions that pass the third level of assessment (M3 level of the pyramid) are reimbursed after approval of their funding request by the National Institute for Health and Disability Insurance (NIHDI). The evidence required for reimbursement of the solutions follows a template (dossier) to assess the care pathway or process related to the app's purpose, i.e., explore the current pathway and how it changes with the use of the app. The reimbursement plan can consider the different verticals (budget lines) of the health payment system. There is currently one application reimbursed, and an agreement has been established between the NIHDI and the healthcare providers (hospitals and physiotherapists) (49).

In France, DTx reimbursement is currently following similar patterns as medical devices, which resembles drug reimbursement (50). Once CNEDiMTS completes the technical review of the actual clinical benefit and clinical added value compared with existing therapies, the Economic Committee for Health Products (CEPS) negotiates the prices to be paid by the statutory insurance system. Manufacturers and CEPS then sign a contract stipulating a price for the therapy and forecasting script volumes. If actual prescription volumes exceed this forecast, manufacturers must rebate between 50 and 80% of additional revenues back to the French government. After these negotiations, the National Union of Health Insurers (UNCAM) registers new therapies on a list of reimbursable products and sets a reimbursement rate that corresponds to therapies' clinical benefits rating: for important benefits, and 100% for major benefits. Since reimbursement rates are set by UNCAM, net price increases do not occur in the 5 years after drugs initially gain market access. By rewarding added value within limits, the French drug pricing system strikes a robust balance between lower prices and innovation. Medical Devices that receive permission to be reimbursed are included in the list of products and services qualifying for reimbursement (List des Produits et Prestations Remboursables—LPP) (51).

In NHS England, there is no direct connection between the DTAC or ESH frameworks certification and reimbursement. A positive endorsement from NICE can support the acceptance and adoption of the health app by providers and Clinical Commissioning Groups (CCGs) (25). CCGs are primary care-led groups that include the GP groups in a particular area and represent statutory NHS bodies responsible for the planning and commissioning of health care services for their area, that might provide reimbursement for a digital technology depending on their strategy. Therefore, DTx solutions may strive to achieve certification by NICE/NHS and be purchased at a national or regional level by CCGs. However, through dedicated programs such as NHS Innovation Accelerator, the NHS selects innovations to be integrated in the health and care system (52).

The Spanish public health system has no defined framework for reimbursement of digital health solutions. The Catalan system is purchasing health products and services through public or pre-commercial procurement (PCP) by launching specific tenders. The current approach for general reimbursement is a top-down approach, their strategy being focused on the reimbursement of the care pathway, and not isolated elements. The current pathway in focus is diabetes, and a tender for diabetes is soon to be published by the main healthcare provider, CatSalut. The tender covers multiple aspects related to diabetes care needs (i.e., glucometers), but also contains requirements for diabetes apps: passing the TICSS Certification Process, providing the CE-certification and proof of possibility of integrating the solution with the mConnecta platform (30). The Catalan evaluation approach is based on assessing how elements can improve the existing pathways in an integrated-care way, considering both the system and the patient. If the solutions are funded through the public tender, they can be integrated into the health system.

With regards to market stimulation, besides Germany, most countries focus on health care improvement rather than market stimulation. Germany is a notable difference as they allow reimbursement with provisional evidence within the first 12 months and relatively free pricing for the initial year, after which the pricing levels are renegotiated.

In terms of how DTx can be procured, there is a need for agreement on the patient-reported outcome data that could be used to determine models of payment (registry based, licensing, reimbursement per use, prescription). Shifts in budget and service allocation are often not considered in price negotiations and should be, from a system perspective, taken into account (e.g., in cases where care is shifted from a hospital to a different organization, team or even to the supplier of DTx). Most of the reimbursement scenarios observed during the study are reimbursement of a novel care pathway that incorporates a DTx, and not a direct reimbursement of the technology solution (except for the German model). One key question that policymakers should consider is whether reimbursement should be made to a single healthcare organization which then has the business justification for a procurement of the DTx. This model has anecdotally been shown to be unfavorable to DTx that support cross-organizational collaborative care, since no one healthcare organization is a complete beneficiary to justify the procurement. On the other hand, not every DTx applies to cross-organizational care and regulation and reimbursement structures should account for this variation.

Another issue from the manufacturers' perspective is that there currently seems to be a lack of dedicated processes and transparent guides for DTx assessment. A solid business case for manufacturers further reduces barriers for DTx development. The model for both traditional therapeutics (i.e., mostly drugs) and DTx requires large investments, making this market feasible only for larger players. To facilitate market access for SMEs and a wider pool for innovation while following strict clinical evidence standards is not an easy equation to solve. Solutions to this challenge could most likely be achieved through public funding programs directed specifically to trialing DTx.

Finally, a step forward toward a promising DTx reimbursement, integration and pricing pathway could be a value-based approach, i.e., payment/reimbursement for additional value added compared to existing practices. However, challenges remain. Value-based models could solve some challenges with DTx as the healthcare providers would have to find solutions to prevent escalation and to work across the current siloes. Value-based healthcare focuses on health outcomes instead of activity (i.e., paying for the number of procedures). This is widely recognized as a promising solution to many health-system challenges but the practical application of it is difficult. The difficulty for DTx companies lies in the lack of direct control over the use of their solutions. The DTx itself can have immense potential but realization of this value depends on the way it is used and the broader way of working at the healthcare provider. One key driver of price is not only the absolute value a product delivers but also its relative value to other existing DTx solutions. Therefore, there will be a future point in time where several DTx solutions are on the market and the demand for any new solutions must carefully be assessed in terms of market competition. This could introduce a soft cap on the amount of DTx for a certain disease type or patient group to prevent health system expenditure from increasing. Existing DTx solutions should also be re-assessed, and their price be adjusted according to performance data which could be obtained from insurance datasets.

### Integration Into Healthcare Systems

Evaluation, certification, and reimbursement are essential steps for the DTx to reach its' end user: the patient. There is scarce information on DTx prescription practices as the phenomenon is rather new. In Germany, DiGAs can currently be prescribed by primary care physicians and psychotherapists. However, hurdles have been identified in relation to the general workflow of the prescription process, as DiGAs are currently prescribed on paper. There is also resistance from German physician organizations to raise awareness on DiGAs. Currently the system works according to a bottom-up approach: developers target directly their customers; the latter find out about the solutions. Patients usually then ask their physicians for a prescription, but alternatively seek direct reimbursement from the statutory health insurance companies. While information campaigns are on-going, a strong impact has not been observed yet. The German government is exploring possibilities on how to incentivize the prescription and use of DiGAs. In Belgium, medical doctors are allowed to prescribe DTx, which are targeted toward broad patient groups as per results of the notification form (53). In UK, DTx solutions can be prescribed by GPs if their CCG/NHS trust groups have commissioned them. In France, DTx included in the LPP can be prescribed by physicians to patients. In Catalonia, the prescription of health apps was piloted and physicians are able to prescribe health apps but without patient reimbursement (54).

Integrating and yielding the most value out of DTx is difficult and requires change management from the health system, and mainly on the engagement of clinicians in how DTx can fit into clinical workflows and practice. There is resistance toward clinical acceptance and organizational change, and issues that have been raised include anxieties amongst clinicians about their professional responsibility for care pathway elements which are placed in the hands of patients, and with certain levels of care guidance being provided by the technology and not by them. Secondly, there is a concern about the investment of time and expertise required to educate patients about how to use the technology and about how to manage those elements of their care which are supported by the technology, including the criteria that should trigger them to escalate a concern to a clinician. Who will pay for this time investment and who has the relevant training to provide it? Will this be an extra burden on each medical practitioner, or will there be enough budget to employ someone who coaches the patients and can support them with any issues at home? Another important resistance factor amongst clinicians is about what reimbursement they will get for elements of care that the technology looks after and therefore the possibilities of financial losses for services that are being replaced by the digital technologies. Ideally, these issues should be researched in more detail with a wider range of health and care professionals.

### Data Integration and Use of DTx Data

Data integration is generally addressed through wider strategies. In France, data integration is expected to be achieved through the integrated approach of the new health data strategy, operationalized by several organizations, and enabled by the national Health Data Hub (HDH) (55). The patients' health data space and the professional one is linked *via* interoperability services by the L'ANS competence center.

In most of the surveyed countries, the approval of a DTx required interoperability with the national Electronic Health Record, eHealth platform, or certain interoperability standards, and sometimes to specific Application Programming Interfaces (APIs). In Belgium for example, interoperability standards compliance is required and verified before approval of the health app as part of a second level of the assessment process, which specifies that if data is to be shared or processed, this should take place via open standards proposed by the eHealth platform (26).

However, most of the studied countries and regions are still exploring how to import the DTx data and to incorporate it as part of the longitudinal health record of each patient. The level of interoperability between different functions of EHR varies among the countries based on different indicators such as the level of usage by different care organizations, the type of data, or characteristics of data exchange. For example, Germany shows more widely a low level of health data exchange e.g., the ePrescription system is still on piloting phase (56). A rather similar case applies for France, as the use of national EHR systems, and the level of usage is slightly higher than that of Germany. Given that most EU countries' national-level EHR systems are in the phase of being rolled-out and interoperability to all care sectors, facilities and practices is not fully established, integration of DTx data into routine care and Health Information Systems (HIS) is and will remain more a vision than reality in the near future.

As such, many DTx operate in their own “data bubble” due to the limits of the health system and its infrastructure consisting mostly of EHRs. An interesting approach to DTx data integration is represented by the interoperable mConnecta platform developed by the Catalan government (30). The platform collects data produced by devices that are not normally collected within the framework of formal healthcare provision services (e.g., EHR). mConnecta stores data from mobile apps, wearables, and medical devices and integrates the generated data with the EHR, and with available data for standard care on primary and hospital settings. At the moment of data collection, the platform was being piloted in two hospitals and two primary care settings.

### Secondary Use of Data and Data Reusability

Secondary use of health data, and DTx data in particular, is a complex topic as the structure of data often hinders effective data use and overly strict data protection laws limit the use and extents of secondary purposes. Several strategies and policies address the secondary use of health data. In the National Data Strategy recently published by UK, secondary use of health data is seen as a top priority, and strategy goals related to use of health data in research have been defined. Currently, health data is used at the NHS level mostly for research and monitoring purposes, but DTx data is not used for this purpose even though there is interest (57). In Belgium, the Data for Better Health Strategy proposes strategic actions and addresses challenges to support secondary use of health data (58). However, the role of the healthdata.be platform, whose mission is to facilitate the data exchange between healthcare professionals and researchers to increase public health knowledge, is unclear with regards to DTx data (59). In France, innovation from health data is facilitated by the newly established Health Data Hub (HDH), which interfaces with data providers and data consumers and through a general, however non-exclusive practice, of entering into contact with data consumers. The HDH handles both personal and anonymized data (4 categories of data: personal for care, personal for research, research under specific conditions, RWD and anonymized data). It also supports the hospitals and other data generating organizations to collect data meeting the quality and interoperability requirements for an eventual multi-purpose use. Hospitals partner with the HDH, and they in return get back the results of the research they contributed to, but also other HDH supported research. Appropriate governance is in place to make sure there is equal access of all industry and full transparency of such access. Industry can get access to data from the HDH if there is a clear and validated protocol for the purpose and way of use. Industry may contribute some budget globally into a fund, reinvested into supporting the functions of the HDH. In Germany, secondary use of DTx data is allowed (40), and secondary use for research purposes upon patient consent will be possible once the technical infrastructure in Germany allows data transfer between the patient's EHR and the Research Data Center (60), the central organization of primary datasets for legitimate research purposes.

None of the countries had yet put in place a formalized approach to the reuse of data originating from DTx, for secondary purposes yet it was usually an aspiration for the health system to do this. Although health data research centers exist, the lack of technical capabilities (interoperability with EHRs, structured data) strongly prevents the effective re-use of data. We did not encounter a scenario in which the developer of the DTx is permitted to commercially utilize the data they collect.

There is an increasing interest, especially from the medical device and Artificial Intelligence (AI) sector, to have access to patient-generated data. Therefore, DTx generated data is a valuable resource. The obstacles to reusing the data seem to lie between legislative restrictions and the implementation of rich enough interoperability and control over the data despite existing standards, methodologies and solutions (see other papers in this volume). There is very little DTx data reuse culture. A major identified benefit of shared DTx data would be decentralized clinical trials where manufacturers obtain (given the consent of all involved patients) real-world datasets which can be used as an evidence base for any impact assessment without conducting patient recruitment. From the patient's perspective, consent management could be improved through support models or platforms to make it as easy as possible to consent to their data being used for specific trials or studies. Another possibility is to integrate patient-generated data into the same approval processes and secondary use models designed for EHR data.

## Key Findings and Recommendations for Enabling Better Integration of DTX Into Healthcare Systems

### Success Factors for Scaling Up DTx Adoption

Navigating the health system, its organizations and understanding its structures can be challenging especially for new market entrants, but also for established players when new frameworks are introduced. While regulating market access is the main responsibility of regulators, there are needs for guidance and clear paths for DTx providers to understand the different market entry options, responsibilities of relevant bodies and the processes toward DTx certification and deployment as well as the steps within these processes. The investigation revealed several factors that could enable rapid uptake of innovation and ensure a better healthcare market access. These main success factors are summarized as recommendations in Table 1 and discussed in the rest of this section.

**Table 1:** Summary of the success factors identified through this research.

**Inclusive national strategies for DTx**
• DTx should be recognized as a key enabling technology and should be included into broader digital health strategies to ensure a harmonized and integrated approach in the digital health ecosystem.
**Regulation for innovation**
• Countries should define a framework and criteria for assessing DTx that optimizes and balances regulation and innovation. • Frameworks needs to consider the adoption process from the provider perspective in addition to the regulatory perspective. • Features of the evaluation procedures should include a publicly available standardized catalog of the required evidence, indicator types and a defined set of accepted methods.
**Clinical evidence**
• Assessment of clinical impacts needs to highlight the necessary changes to care processes and new interactions between care stakeholders. • The required evidence should be aligned with the requirements stipulated in the European Medical Device Regulation, to create a portfolio of evidence that is valid and relevant across the EU. • Assessment frameworks should provide temporary reimbursement for a CE marked DTx to enable placing in the market and use of the solution by patients and clinicians for a limited period of time, so DTx providers can gather real-world evidence to support clinical and health economic evaluation.
**Socio-economic evidence**
• Changes to the way of clinical practice and workflow in general need to be considered and pose opportunities for extending the cost-efficiency of the DTx itself. • The healthcare system should provide necessary information, especially on health systems costs, to the DTx provider, for the benefit of both parties.
**Additional assessment criteria**
• DTx are patient-facing, therefore additional criteria for interoperability, privacy, and security by design, on top of Medical Device Regulation certification, as well as usability and accessibility criteria should be explicitly specified.
**Clear-cut assessment and certification pathways for DTx solutions linking approval and reimbursement**
• There should be a clear link between DTx certification and reimbursement, and namely, certification should trigger rapid price setting and a reimbursement mechanism. • HTA pathways for DTx solutions should be established, together with clear guidelines, requirements, and information on the process (e.g., length of processing and regular status updates).
**Fostering innovation through the DTx industry**
• High research costs can partially be leveled through public funding during the data generation phase, and national innovation programmes should be put in place to encourage partnerships between industry and health care providers to work together. • Manufacturers should be offered possibilities to generate data from real patients, where clinical pathways can appropriately accommodate the DTx innovation, while reimbursing the solution at an appropriate level. • The risks for public payers can be managed by requiring strict scrutiny for entry but at the same time allowing providers to discover the optimal ways of working under real clinical conditions. • Accompanying guidelines for providers significantly improves the transparency of the acceptance process, reduce the business risks for providers and are expected to stimulate innovation. • DTx frameworks should aid small and large companies already in the phase of data generation through initial financial support (through funds or commercial revenue in combination with an initial marketing authorization). • New agile business models, such as pay for use, or the prescription of apps, could be valid and useful alternatives to current licensing-based revenue streams for companies. • Payers interested in such models may consider implementing dynamic ways to adjust price and reimbursement levels. This can be achieved, for instance, by basing the price on volumes of usage or reimbursement after a defined period of use by patient.
**Strategies for change management and capacity building for the involved stakeholders**
• Change resistance can be managed on a general level by increasing the understanding of the potential impacts of DTx and by transparently communicating the (desired) changes and their implications. • Information of the approvals and assessment of DTx should be visible to all stakeholders involved in decision making and potential adoption. • Enable patients and clinicians to decide on the relevance of a DTx for their individual needs by facilitating the comparison of DTx solutions for specific conditions or within a certain care pathway, based on quality criteria that they can easily understand.
**Secondary use of DTx data and use of RWE data**
• Decentralized clinical trials could offer manufacturers the possibility to use real-world datasets to generate evidence for impact assessment without having to recruit trial patients. • Consent management could be improved through support models or platforms. • Whilst secondary data use is probably not a direct success factor for a DTx developer, this secondary use can be a success factor for learning from the data to better design and implement more personalized care.

#### Inclusive National Strategies for DTx

Currently, DTx solutions are not part of national or regional strategies but rather only parts of certain innovation programs or laws, which contributes to lower uptake of DTx solutions. Given the complexity of the solutions, DTx should be recognized as such and should be included into broader digital health strategies to ensure a harmonized and integrated approach in the digital health ecosystem.

#### Regulation for Innovation

Countries should define a framework and criteria for assessing DTx that optimizes and balances regulation and innovation. Many such frameworks are publicly available both from official national frameworks, trade organizations (e.g., DTxAlliance), and working groups (e.g., EUnetHTA), on which governments can build.

A DTx framework needs to consider the process from the provider perspective in addition to the regulatory perspective. Key aspects to be addressed are transparency (of the process and of the criteria) and efficiency. Requirements, processing time and status of the application should be clear and visible, preferably accessible online. The process from submitting the application to certification should not take more than a few months at maximum.

For DTx providers, the one key support feature of any evaluation and assessment procedure is a publicly available standardized catalog of the required evidence, indicator types and a defined set of accepted methods. Compared to the wide field of medical device assessment, a more streamlined and speedier process is overall preferred to enable the innovative potential of any DTx solution. Recognized methods such as Randomized Controlled Trials, Cost-Benefit Analysis and Cost Effectiveness Analysis are regarded as appropriate and rigorous tools for the generation of evidence, but other forms of evidence should be accepted depending on the specifics.

#### Clinical Evidence

In terms of clinical evidence generation, demonstration of improved quality of care and better clinical outcomes through DTx is the desirable goal. Clinical trials are an industry-standard with long-standing acceptance and scientific robustness and can be regarded as the gold-standard for quantifying clinical impacts. However, DTx fail to achieve their full potential if they remain isolated within existing care pathways. Assessment of clinical impacts will however need to also highlight the necessary changes to care processes and new interactions between stakeholders. The exact type of required evidence can be aligned to the requirements stipulated in the European Medical Device Regulation to create a set of evidence that is valid and relevant also across countries. A potential solution to balance clinical evidence and real-world results is to offer provisional acceptance period based on clinical evidence. This period can be utilized by the DTx provider to gather information on the real-world effects.

#### Socio-economic Evidence

For socio-economic evidence health systems and healthcare providers need to provide data and support to DTx providers to achieve the best results for all parties. Using general cost estimates does not suffice when comparing DTx to an existing and specific care pathway. Changes to the way of clinical practice and workflow in general need to be considered and pose opportunities for more cost-efficiency. The obligation to furnish this evidence, including the cost of its production, is always to be borne by the developer. On the other hand, available socioeconomic data collected for other primary purposes (e.g., reimbursement of health care) is not always of suitable quality for the purposes of DTx assessment. A national framework could provide temporary reimbursement for a CE marked DTx to enable placing in the market and use of the solution by patients and clinicians for a limited period of time, while real data can be collected to support clinical evaluation. The healthcare system should provide necessary information, especially on costs to the DTx provider for the benefit of both parties.

#### Additional Criteria

DTx are patient-facing, therefore additional criteria for interoperability, privacy, and security by design, on top of Medical Device Regulation certification as well as usability and accessibility criteria should be explicitly specified. For all of these, industry standards (such as ISO 82304-2 for health software) do exist, and they provide the blueprint for these requirements (61).

#### Clear-Cut Assessment and Certification Pathways for DTx Solutions Linking Approval and Reimbursement

DTx providers use several business models to commercialize their products, including license agreements and direct negotiation with hospitals. Current HTA pathways are rather slow and complicated to navigate and do not necessarily lead to reimbursement for DTx products. This makes it difficult for developers to scale up, is unfriendly to new market players, and inhibits innovation. To facilitate rapid uptake of innovation, there should be a clear link between DTx certification and reimbursement, and namely, certification should trigger rapid price setting and a reimbursement mechanism.

HTA pathways for DTx solutions should be established, together with clear guidelines, requirements, and information on the process (e.g., length of processing and status). Depending on the specifics of the country, a process analogous to pharmaceuticals may be sensible, but this process should be much more streamlined and shorter than with pharmaceuticals and should focus on the effects of specific DTx within the care pathways they are designed to be applied in. Experts remarked that re-using pathways for pharmaceuticals for DTx can endanger DTx, if the existing shortcomings of pharmaceutical pathways are transferred to DTx pathways.

#### Fostering Innovation Through the DTx Industry

The costs (patient recruitment costs, personnel costs for long trials, development costs) significantly raise product development costs and may discourage potential DTx providers. High research costs can partially be leveled through public funding during the data generation phase, and national innovation programs may be put in place to encourage partnerships between industry and health care providers to work together on ICT enabled re-engineering of clinical processes and demonstrating the value of the DTx innovation at hand.

An alternative approach would be offering manufacturers a possibility to generate data from real patients, where clinical pathways can appropriately accommodate the DTx innovation, while reimbursing the solution at an appropriate level. The risks for public payers can be managed by requiring strict scrutiny for entry but allowing providers to discover the optimal ways of working under real clinical conditions. The German DiGA model with its real-world evidence process, where the manufacturer receives initial approval and reimbursement for a year to collect additional data, is one example of balancing risks, level of evidence and overall duration of the assessment procedure. Accompanying guidelines for providers significantly improve the transparency of the process, reduce the business risks for providers and are expected to stimulate innovation.

Companies have been developing and marketing DTx solutions as Medical Devices for many years and are expected to continue to do so. However, the introduction of new and more lucrative business models and market entry pathways have a great potential to spur innovation. As such, DTx frameworks should aid small and large companies already in the phase of data generation through initial revenue (through funds or commercial revenue in combination with an initial marketing authorization). This step would lower the bar for smaller companies to enter the market with less capital and provides an incentive to enter a clinical and economic evaluation and assessment process which requires thorough and costly data collection methods (such as RCTs).

New agile business models, such as pay for use, or the prescription of apps could be a solid alternative to current licensing-based revenue streams for companies. Payers interested in such models may consider implementing dynamic ways to adjust price and reimbursement levels. This can be achieved, for instance, by basing the price on volumes of usage or reimbursement after a defined period of use by patient.

#### Strategies for Change Management and Capacity Building for the Involved Stakeholders

Traditional healthcare systems, with their complex networks of stakeholders and responsibilities, have developed a certain resistance to radical change. This should be considered (with sensitivity but as an obstacle) when conceiving new frameworks, even though change itself cannot be avoided. However, change resistance can be managed on a general level by increasing the understanding of the potential impacts of DTx and by transparently communicating the (desired) changes and their implications. This is a task for both the regulators and payers, and for DTx providers. Clinicians broadly trust published studies and data on drug effectiveness and treatment risks. Information of the approvals and assessment of DTx should be visible to clinicians and handled in a similar manner.

Patients are expected to adapt quickly to changes introduced by DTx, provided that they can trust them. Health and wellness apps already play a significant role in mHealth, while there currently is a lagging of DTx in the healthcare system. One way for enabling patients and clinicians to decide on the relevance of a DTx for their individual needs would be to filter and compare DTx solutions for specific conditions or within a certain care pathway, based on quality criteria that they can easily understand.

#### Secondary Use of DTx Data and Use of RWE Data

Data collected by DTx is not fully harnessed in Europe. Although in certain cases it is planned, no DTx data is used in the health system for other uses than the primary use within the DTx, although strategies for the secondary use of health data have been defined. Real-world evidence is an interesting topic, but its potential is mostly left unused. Better access to data bears huge potential to realizing additional benefits for businesses and governments. Decentralized clinical trials could offer manufacturers the possibility to use real-world datasets to generate evidence for impact assessment without having to recruit patients and consent management could be improved through support models or platforms. Whilst secondary data use is probably not a direct success factor for a DTx developer, this secondary use can be a success factor for learning from the data to better design and implement more personalized care. This source of valuable data might incentivize health systems to promote wider DTx adoption.

### Strengths and Limitations

The approach to the investigation sought a thorough analysis of the current situation regarding adoption of digital therapeutics in five selected countries and regions.

The investigation included interviews with experts with intimate knowledge of relevant national and regional DTx efforts. In most cases, the interviewees were representatives of the bodies responsible for running or setting up DTx national or regional programs, or representatives of DTx providers who were or are planning to take part in those programs with DTx solutions they have been developing. However, the topic of digital therapeutics is a fast-moving one, and despite best efforts, it may be possible that brand-new developments are not considered. Some of the interviewed experts might be unfamiliar with other relevant initiatives within their national systems. For example, only recently did President Emmanuel Macron announce his desire for France to replicate the German DIGA approach, the implications of which are only now starting to be revealed. DTx adoption and research into good national and regional practices is an exciting area which requires further attention in the years to come.

Pertinent good practices and examples may be available in other European countries, and some of them have been communicated to the investigators, e.g., through the multi-stakeholder expert workshop which hosted experts from many EU countries and who reflected on their own national experiences. While many expert inputs at the workshop confirmed the general conclusions of the investigation as well as the identified success factors and barriers to DTx adoption, the investigation cannot generalize its conclusions and recommendations across Europe, since they have only been derived from the five investigated countries and regions. Further work is needed to validate these findings and success factors more broadly across Europe, although the authors suspect many of them findings will be generally applicable.

### Recommendations for Future Work

The findings in this paper are preliminary and based on a limited sample of countries and experts per country. It may be noted that these success factors and potential recommendations to decision makers are more specific to the context of DTx adoption than the general success factors for digital health adoption that were identified from the literature reported earlier. Our findings should be validated by wider range of stakeholders: a greater number and diversity of stakeholders from more European countries. We believe that the success factors found through our research represent the main factors on a high level. The practical approaches to these factors should be investigated further. This field is advancing quickly, and the value and feasibility of different models will be tested in the coming years. Effort should be placed on cross-country recognition of evidence and certification.

## Data Availability Statement

The raw data supporting the conclusions of this article will be made available by the authors, without undue reservation.

## Ethics Statement

Ethical review and approval was not required for the study on human participants in accordance with the local legislation and institutional requirements. The patients/participants provided their written informed consent to participate in this study.

## Author Contributions

All authors listed have made a substantial, direct, and intellectual contribution to the work and approved it for publication.

## Funding

SITRA funded a study on European approaches to digital therapeutics. I-HD and empirica were the suppliers for the study, as part of the competitiveness from health data project of the SITRA's Health data 2030 program. The material gathered in that study was used in this paper. This paper was not funded by SITRA.

## Conflict of Interest

The authors declare that the research was conducted in the absence of any commercial or financial relationships that could be construed as a potential conflict of interest.

## Publisher's Note

All claims expressed in this article are solely those of the authors and do not necessarily represent those of their affiliated organizations, or those of the publisher, the editors and the reviewers. Any product that may be evaluated in this article, or claim that may be made by its manufacturer, is not guaranteed or endorsed by the publisher.

## References

[1] Marques IC Ferreira JJ. Digital transformation in the area of health: systematic review of 45 years of evolution. Health Technol. (2020) 10:575–86. 10.1007/s12553-019-00402-8

[2] Kraus S Schiavone F Pluzhnikova A. Invernizzi AC. Digital transformation in healthcare: Analyzing the current state-of-research. J Bus Res. (2021) 123:557–67. 10.1016/j.jbusres.2020.10.030

[3] Dtxalliance.ORG. UnderstandingDTx. In. (2021). Available online at: https://dtxalliance.org/understanding-dtx/

[4] Sverdlov O Van dam J Hannesdottir K Wells TT. Digital therapeutics: an integral component of digital innovation in drug development. Clin Pharmacol Ther. (2018) 104:72–80. 10.1002/cpt.1036 29377057

[5] Lauer W Löbker W Höfgen B. Digital health applications (DiGA): assessment of reimbursability by means of the “DiGA Fast Track” procedure at the Federal Institute for Drugs and Medical Devices (BfArM). Bundesgesundheitsblatt Gesundheitsforschung Gesundheitsschutz. (2021) 64:1232–40. 10.1007/s00103-021-03409-7 34529095 PMC8492566

[6] Yan K Balijepalli C Druyts E. The impact of digital therapeutics on current health technology assessment frameworks. Front Digital Health. (2021) 3:1–3. 10.3389/fdgth.2021.667016 34713140 PMC8521991

[7] Blobel B Ruotsalainen P. Healthcare Transformation Towards Personalized Medicine-Chances and Challenges. Stud Health Technol Inform. (2019) 261:3–21. 31156085

[8] Lovell T. NHS Scotland Provides Access to Digital Therapeutics for Anxiety and Insomnia. HIMSS Media (2021).

[9] DieTechniker. Gesund-heits-Apps auf Rezept: Das DiGA-Verzeichnis und der Antragsweg (2021). Available online at: https://www.tk.de/techniker/gesundheit-und-medizin/kompetent-als-patient/gesundheits-apps-auf-rezept-2084828

[10] Powell AC Torous JB Firth J Kaufman KR. Generating value with mental health apps. BJPsych Open. (2020) 6:e16. 10.1192/bjo.2019.98 32019619 PMC7176837

[11] Rodriguez-villa E Torous J. Regulating digital health technologies with transparency: the case for dynamic and multi-stakeholder evaluation. BMC Med. (2019) 17:1–5. 10.1186/s12916-019-1447-x 31801532 PMC6894205

[12] Rahimi K. Digital health and the elusive quest for cost savings. Lancet Digit Health. (2019) 1:e108–9. 10.1016/S2589-7500(19)30056-1 33323258

[13] Wolff J Pauling J Keck A Baumbach J. The economic impact of artificial intelligence in health care: systematic review. J Med Internet Res. (2020) 22:e16866. 10.2196/16866 32130134 PMC7059082

[14] Yamey G. Scaling up global health interventions: a proposed framework for success. PLoS Med. (2011) 8:e1001049. 10.1371/journal.pmed.1001049 21738450 PMC3125181

[15] Mhealthhub. d2.1 Knowledge Tool 1. Health Apps Assessment Frameworks (2020). Available online at: https://mhealth-hub.org/download/d2-1-knowledge-tool-1-health-apps-assessment-frameworks

[16] Labrique AB Wadhwani C Williams KA Lamptey P Hesp C Luk R. Best practices in scaling digital health in low and middle income countries. Global Health. (2018) 14:1–8. 10.1186/s12992-018-0424-z 30390686 PMC6215624

[17] Desveaux L Soobiah C Bhatia RS Shaw J. Identifying and overcoming policy-level barriers to the implementation of digital health innovation: qualitative study. J Med Internet Res. (2019) 21:e14994. 10.2196/14994 31859679 PMC6942191

[18] Lennon MR Bouamrane MM Devlin AM O'Connor S O'Donnell C Chetty U. Readiness for delivering digital health at scale: lessons from a longitudinal qualitative evaluation of a national digital health innovation program in the United Kingdom. J Med Internet Res. (2017) 19:e6900. 10.2196/jmir.6900 28209558 PMC5334516

[19] Marwaha JS Landman AB Brat GA Dunn T Gordon WJ. Deploying digital health tools within large, complex health systems: key considerations for adoption and implementation. NPJ Digit Med. (2022) 5:1–7. 10.1038/s41746-022-00557-1 35087160 PMC8795422

[20] Van velthoven MH Cordon C. Sustainable adoption of digital health innovations: perspectives from a stakeholder workshop. J Med Internet Res. (2019) 21:e11922. 10.2196/11922 30907734 PMC6452285

[21] Pikkarainen M Hyrkäs E Martin M. Success factors of demand-driven open innovation as a policy instrument in the case of the healthcare industry. J Open Innov Technol Mark Complex. (2020) 6:39. 10.3390/joitmc6020039

[22] Schlieter H Marsch LA Whitehouse D Otto L. Scaling-up digital innovations in healthcare: expert commentary on success factors and barriers. J Med Internet Res. (2019) 21:e14994. 10.2196/24582 35275065 PMC8956989

[23] Bfarm. DiGA. In. (2021). Available online at: https://www.bfarm.de/DE/Medizinprodukte/Aufgaben/DiGA/_node.html

[24] EHEALTH.FGOV.BE. Roadmap 3.0. In. (2019). Available online at: https://www.ehealth.fgov.be/nl/egezondheid/roadmap-30

[25] Maniatopoulos G Haining S Allen J WILKES S. Negotiating commissioning pathways for the successful implementation of innovative health technology in primary care. BMC Health Serv Res. (2019) 19:1–12. 10.1186/s12913-019-4477-3 31492139 PMC6731596

[26] MHEALTHBELGIUM. Validation Pyramid - mHealthBELGIUM. In. (2020). Available online at: https://mhealthbelgium.be/validation-pyramid

[27] NHSX. Digital Technology Assessment Criteria (DTAC). In. (2022). Available online at: https://www.nhsx.nhs.uk/key-tools-and-info/digital-technology-assessment-criteria-dtac/

[28] NICE. Evidence Standards Framework for Digital Health Technologies. In. (2021). Available online at: https://www.nice.org.uk/about/what-we-do/our-programmes/evidence-standards-framework-for-digital-health-technologies 10.1177/20552076211018617 PMC8236783 34249371

[29] Santé HAD,. Medical Device Health Technology Evaluation Committee (CNEDiMTS*). In. (2019). Available online at: https://www.has-sante.fr/jcms/c_2036238/en/medical-device-and-health-technology-evaluation-committee-cnedimts

[30] MHEALTH-HUB. mConnecta Platform. In. (2021). Available online at: https://mhealth-hub.org/mconnecta-platform

[31] MHEALTH.CAT. Mobile Health Plan. In. (2021). Available online at: https://smartcatalonia.gencat.cat/web/en/projectes/govern/detalls/article/Pla-de-mobilitat-mHealth.cat

[32] Health MOSA. National Health Strategy 2018 – 2022. (2017). Available online at: https://www.moph.gov.qa/english/strategies/National-Health-Strategy-2018-2022/Pages/default.aspx

[33] BGBL. Gesetz für eine bessere Versorgung durch Digitalisierung und Innovation (Digitale-Versorgung-Gesetz – DVG. In. (2019) Nr.49:2564–83.

[34] London Department of Health & Social Care. Official Home Page. In. (2021). Available online at: https://www.gov.uk/government/organisations/department-of-health-and-social-care

[35] NHS. NHS. Accelerator. In. (2021). Available online at: https://nhsaccelerator.com/

[36] NHS. Accelerated Access Collaborative. In. (2021). Available online at: https://www.england.nhs.uk/aac/

[37] MHEALTHBELGIUM. Official Home Page. In. Available online at: https://mhealthbelgium.be/

[38] NICE. Digital Health Technologies. In. (2021). Available online at: https://www.nice.org.uk/about/what-we-do/digital-health

[39] UNION E,. Regulation (Eu) 2017/745 of the European Parliament of the Council of 5 April 2017 on Medical Devices, Amending Directive 2001/83/EC, Regulation (EC) No 178/2002 Regulation (EC) No 1223/2009 Repealing Council Directives 90/385/EEC 93/42/EE. In. (2017). Available online at: https://eur-lex.europa.eu/legal-content/EN/TXT/PDF/?uri=CELEX:32017R0745

[40] DIGAV. Verordnung über das Verfahren und die Anforderungen zur Prüfung der Erstattungsfähigkeit digitaler Gesundheitsanwendungen in der gesetzlichen Krankenversicherung (Digitale GesundheitsanwendungenVerordnung - DiGAV). In. (2020). Available online at: https://www.gesetze-im-internet.de/digav/BJNR076800020.html

[41] DIGA. DiGA-Verzeichnis. In. (2021). Available online at: https://diga.bfarm.de/

[42] NHSX. Digital Technology Assessment Criteria. Available online at: https://www.nhsx.nhs.uk/key-tools-and-info/digital-technology-assessment-criteria-dtac/

[43] TICSALUTSOCIAL. mHealth. In. (2021). Available online at: https://ticsalutsocial.cat/el-tipus/mhealth/

[44] Santé HAD,. Guide to the Specific Features of Clinical Evaluation of a Connected Medical Device (CMD) in View of Its Application for Reimbursement (2019). p. 7–18. Available online at: https://www.has-sante.fr/upload/docs/application/pdf/2021-08/guide_to_the_specific_features_of_clinical_evaluation_of_cmd_in_view_of_its_application_for_reimbursement.pdf

[45] Santé HAD,. Assessment Principles Established by the Medical Device Health Technology Evaluation Committee (CNEDiMTS) to Determine the Reimbursement Eligibility of Medical Devices for Individual Us. (2019). p. 6–28. https://www.has-sante.fr/upload/docs/application/pdf/2019-11/assessment_principles_established_by_cnedimts.pdf

[46] RIZIV. Fabrikanten en verdelers van mobiele medische toepassingen: uw applicatieaanmelden. In. (2021). Available online at: https://www.riziv.fgov.be/nl/professionals/individuelezorgverleners/verstrekkers-van-implantaten/Paginas/fabrikanten-verdelers-medische-mobiele-toepassingen-aanmelden.aspx

[47] TICSS. CertificationFramework. In. (2021). Available online at: https://ticsalutsocial.cat/en/noticia/the-national-security-framework-in-public-procurement/

[48] BFARM. The Fast-Track Process for Digital Health Applications (DiGA) According to Section 139e SGB V. In. (2021). Available online at: https://www.bfarm.de/SharedDocs/Downloads/EN/MedicalDevices/DiGA_Guide.html

[49] NIHDI. Care by the Physiotherapist, Cost and Reimbursement. In. (2021). Available online at: https://www.inami.fgov.be/fr/professionnels/sante/kinesitherapeutes/Pages/default.aspx

[50] Pesqué R Percheron R Cordonnier A Steelandt J. Health technology assessment of innovative medical devices: timing and decision at national and local level. Ann Pharm Fr. (2019) 8:189–97. 10.1016/j.pharma.2019.10.002 31806152

[51] NOMENCLATURES. LPP. In. (2021). Available online at: https://www.ameli.fr/etablissement/exercice-professionnel/nomenclatures-codage/lpp

[52] ACCELERATOR NI,. Official Home Page. In. (2021). Available online at: https://nhsaccelerator.com/

[53] INAMI. Mobile Application Template. In. (2021). Available online at: https://www.inami.fgov.be/SiteCollectionDocuments/formulaire_notification_applications_mobiles_medicales.docx

[54] Lopez segui F Pratdepadua bufill C Rius soler A de san pedro M. Prescription and Integration of accredited mobile apps in catalan health and social care: protocol for the appsalut site design. JMIR Res Protoc. (2018) 7:e11414. 10.2196/11414 30578234 PMC6320420

[55] HDH. Health Data Hub. In. (2021). Available online at: https://www.health-data-hub.fr/

[56] European Commission,. Interoperability of Electronic Health Records. In. (2021). Available online at: https://digital-strategy.ec.europa.eu/en/policies/electronic-health-records

[57] UK department for digital C, media &, sport,. National Data Strategy. In. (2020). Available online at: https://www.gov.uk/government/publications/uk-national-data-strategy/national-data-strategy

[58] Dataforbettterhealth. Data for better health - A Belgian federal government initiative. In. (2021). Available online at: https://dataforbetterhealth.be/

[59] SCIENSANO. Healthdata.be. In. (2021). Available online at: https://www.sciensano.be/en/about-sciensano/sciensanos-organogram/healthdatabe

[60] Forschungsdatenzentrum. Research Data Centre of the Federal Statistical Office. In. (2021). Available online at: https://www.forschungsdatenzentrum.de/en

[61] ISO. ISO/TS 82304-2:2021 Health Software — Part 2: Health and Wellness Apps — Quality and Reliability. In. (2021). Available online at: https://www.iso.org/standard/78182.html

